# Effect of feeding guava agroindustrial waste on nutrient utilization, lamb performance and economic analysis

**DOI:** 10.5194/aab-67-541-2024

**Published:** 2024-11-13

**Authors:** Priscila Torres Nobre, Roberto Germano Costa, Tairon Pannunzio Dias-Silva, Neila Lidiany Ribeiro, Antonio Leandro Chaves Gurgel, Leo Gustavo Coutinho Beltrão, George Rodrigo Beltrão Cruz, Iran Borges, Jose Manuel Lorenzo

**Affiliations:** 1 Departamento de Zootecnia, Universidade Federal da Paraíba, Areia, 58397-000, Brazil; 2 Campus Professora Cinobelina Elvas, Universidade Federal do Piauí, Bom Jesus, 64900-000, Brazil; 3 Instituto Nacional do Semiárido, Campina Grande, 8434-700, Brazil; 4 Escola de Veterinária, Universidade Federal de Minas Gerais, Belo Horizonte, 31270-901, Brazil; 5 Centro Tecnológico de la Carne de Galícia, Parque Tecnológico de Galícia, San Cibrao das Viñas, Ourense, Spain

## Abstract

Our study sought to investigate the impact of incorporating guava agroindustrial waste (GAW) at varying concentrations on nutrient utilization, lamb performance and economic viability. Forty non-castrated, 4-month-old Santa Inês lambs with an initial mean weight of 21.3 ± 2.18 kg were utilized in a confined setting. These lambs were allocated randomly to five treatment groups, each consisting of eight replicates, and were subjected to diets containing increasing levels of GAW (0 %, 7.5 %, 15 %, 22.5 % and 30 % based on dry matter). The feed formulation also included Tifton hay, corn, soybean and mineral salt. Throughout the trial period, parameters such as dry matter intake (DMI), nutrient digestibility and economic metrics were meticulously evaluated. Notably, the performance metrics among the different GAW treatments did not exhibit statistically significant differences ( p > 0.05), with an average daily gain of 328 $g\;d^{-1}$ observed across all the groups. However, there was a discernible positive effect on the intake of ether extract ( p > 0.05). Regarding nutrient digestibility, only the crude protein digestibility demonstrated no significant variance ( p > 0.001) among the treatments. Consequently, our findings suggest that GAW can be effectively integrated into sheep diets at levels of up to 30 % without compromising lamb performance. Moreover, from an economic standpoint, this incorporation proves to be the most financially viable and cost-effective option among the evaluated treatments.

## 1 Introduction

In the quest to mitigate expenses and bolster sheep production, exploring alternative feed sources emerges as a viable strategy, given that feed costs typically constitute approximately 50 % of the overall expenditure in feedlot operations (Santos et al., 2011). Consequently, considerable attention has been directed towards investigating the potential utilization of agribusiness residues within dietary formulations. Notably, guava agroindustrial waste (GAW) has garnered significant interest in this regard. Comprised primarily of pulp with substantial quantities of seeds, GAW boasts noteworthy levels of unsaturated fatty acids and fibrous constituents (Uchôa-Thomaz et al., 2014). Furthermore, studies indicate that GAW exhibits a protein content of approximately 9 % (Bernardino-Nicanor et al., 2006).

Given its chemical composition, GAW exhibits promising potential as a component in animal feed formulations. Its utilization not only holds the prospect of reducing feed costs but also offers the opportunity to impart bioactive compounds, thereby potentially enhancing product quality and promoting consumer health. Parts of the guava tree, such as the leaves and GAW, are rich in antioxidant compounds. In a study conducted with broiler chickens, the addition of guava leaves showed potent antioxidant and antimicrobial activity (Adeyemi et al., 2022). Silva et al. (2014) conducted a study wherein they observed that GAW could be incorporated into sheep diets at substitution levels of up to 40 % without detrimentally impacting productive and carcass performance while maintaining the economic viability of the system. However, at a substitution level of 60 %, the diet exhibited diminished nutrient availability alongside an accelerated rate of digesta passage through the gastrointestinal tract.

Although some studies with sheep have reported a reduction in dry matter intake, nutrient digestibility and average daily gain (Silva et al., 2014), there is still no available information on the economic efficiency of adding GAW to their diet. Thus, we hypothesize that incorporating up to 30 % GAW into the diet of confined sheep would not impair performance but would reduce costs and enhance the economic viability of the production system.

When exploring the utilization of agroindustrial by-products as animal feed, a comprehensive understanding of both productive and economic performance metrics is imperative for assessing dietary efficacy (Silva et al., 2016). Various factors, including animal health (Costa et al., 2018), diet digestibility, feed conversion efficiency (Geron et al., 2015), growth parameters and meat quality (Nobre et al., 2020), play pivotal roles in influencing animal performance outcomes. Our study aimed to evaluate the effects on nutrient intake, digestibility, performance and economic viability from incorporating increasing levels of dehydrated agroindustrial guava by-products into the diet of confined sheep.

## 2 Material and methods

### 2.1 Ethical considerations, experiment location and animals

This study received ethical approval from the Animal Ethics Committee of the Federal University of Paraíba (UFPB), Brazil, under protocol no. 2305/14. The experiment was conducted at the Federal University of Paraíba, specifically at the Bananeiras campus in Paraíba, Brazil, with an altitude of 552 m , latitude 6°41^′^11^′′^ and longitude 35°37^′^41^′′^. Throughout the experiment, the air temperature, measured as black globe temperature, was recorded at 24.97 °C , while the relative humidity within the stalls averaged 76.48 %.

Forty non-castrated Santa Inês lambs, 4 months old and with an average initial weight of 21.33 ± 2.62 kg , were distributed in a completely randomized design in five treatments with eight replicates each and fed increasing GAW levels (0 %, 7.5 %, 15 %, 22.5 % and 30 % based on dry matter). The experiment lasted 63 d , 15 of which were for adaptation to the diets, facilities and management. The animals were identified, weighed and treated for ectoparasites and endoparasites. They were then randomly allocated to slatted and suspended individual stalls (1.50 $m^{2}$ ) and equipped with feeding, drinking and salt troughs.

**Table 1:** Percentage and bromatological composition of experimental diets.

Ingredient ( $g\;{\mathrm{kg}}^{-1}\;\mathrm{DM}$ )	Levels of inclusion (%)				
	0.00	7.50	15.0	22.5	30.0
Guava agroindustrial waste (GAW)^a^	0.00	75.0	150	225	300
Tifton hay	500	425	350	275	200
Ground corn	310	310	310	310	310
Soybean meal	170	170	170	170	170
Mineral supplement^b^	15.0	15.0	15.0	15.0	15.0
Calcitic limestone	5.00	5.00	5.00	5.00	5.00
Chemical composition					
Dry matter, DM ( $g\;{\mathrm{kg}}^{-1}$ as feed)	888	888	889	889	890
Crude protein, CP ( $g\;{\mathrm{kg}}^{-1}\;\mathrm{DM}$ )	154	154	154	154	154
Ether extract, EE ( $g\;{\mathrm{kg}}^{-1}\;\mathrm{DM}$ )	31.3	37.9	44.5	51.1	57.7
Neutral detergent fiber, NDF ( $g\;{\mathrm{kg}}^{-1}\;\mathrm{DM}$ )	489	484	479	474	468
Acid detergent fiber, ADF ( $g\;{\mathrm{kg}}^{-1}\;\mathrm{DM}$ )	249	262	276	290	304
Ash ( $g\;{\mathrm{kg}}^{-1}\;\mathrm{DM}$ )	64.5	60.3	56.2	52.0	47.8
Total carbohydrates, TC ( $g\;{\mathrm{kg}}^{-1}\;\mathrm{DM}$ )	748	744	741	766	734
Non-fibrous carbohydrates, NFC ( $g\;{\mathrm{kg}}^{-1}\;\mathrm{DM}$ )	258	260	262	292	265
Total tannins, TT ( $g\;{\mathrm{kg}}^{-1}\;\mathrm{DM}$ )	0.00	5.00	9.90	14.9	19.8
Lignin^c^ ( $g\;{\mathrm{kg}}^{-1}\;\mathrm{DM}$ )	33.7	44.4	55.0	65.7	76.3
Metabolizable energy, ME (Mcal ${\mathrm{kg}}^{-1}\;\mathrm{DM}$ )	2.48	2.44	2.39	2.34	2.30

^a^ GAW composition: DM – 908; CP – 91.8; EE – 107; NDF – 730; ADF – 620; ash – 21.3; TC – 779; NFC – 48.7; tannin – 6.60 %. ^b^ Composition of the mineral supplement per kilogram: P : 70.0 g ; Ca : 140 g ; Na : 148 g ; S : 12 g ; Mg : 1.32 mg ; F : 700 mg ; Zn : 4.70 mg ; Mn : 3.69 mg ; Fe : 2.20 mg ; Co : 140 mg ; I : 61.0 mg ; Se : 15.0 mg ; monensin: 100 mg . ^c^ Lignin (19.7 %).

### 2.2 Experimental diets

Tifton 85 hay (*Cynodon dactylon* L.) was substituted with dehydrated and ground GAW across varying levels, i.e., 0 %, 7.5 %, 15 %, 22.5 % and 30 %, calculated based on the dry matter (DM) content of the diet. The remaining constituents of the diets comprised ground corn, soybean meal and a vitamin and mineral supplement, all added in equivalent proportions (Table 1). The GAW, predominantly consisting of seeds, was generously provided by Palmeiron (Belo Jardim – Brazil). Dehydration was achieved through sun drying until an approximate moisture content of 10 % was attained. Subsequently, the dehydrated GAW underwent grinding to ensure uniformity within the ration and optimize nutrient availability.

The dietary formulation adhered to a forage-to-concentrate ratio of 50 : 50, aiming to achieve a daily weight gain of 250 $g\;d^{-1}$ as per the guidelines outlined by the NRC (2007). The formulation targeted a crude protein content of approximately 15 % and provided approximately 2.40 kcal of metabolizable energy per kilogram of the diet. The experimental diet, prepared as a total mixed ration, was offered ad libitum to the animals at 07:30 and 16:30 GMT - 3.

### 2.3 Intake and performance

Feed provision and subsequent leftover measurements were conducted daily to facilitate the calculation of voluntary intake and the adjustment of offered quantities, with an allowance of 10 % leftovers based on DM. To estimate nutrient intake, the mean discrepancy between the total nutrient content of the diet offered and that of the leftovers was utilized. Dry matter intake was assessed relative to both live weight (LW) and metabolic weight ( ${\mathrm{MW}}^{0.75}$ ).

Weekly weighings were conducted to monitor the lambs' weight gain. Prior to each weighing session, the animals underwent a 12 h fasting period from solid feed. Total weight gain (TWG) was determined from the difference between the final and initial body weights, while average daily gain (ADG) was calculated by dividing TWG by the duration of the confinement period. The feed conversion ratio was computed as the ratio of cumulative dry matter intake (DMI) (in grams per day) to average daily gain (in grams per day). Additionally, at the conclusion of the experiment, the final live weight (FLW) and average live weight (ALW) of the lambs were recorded over the experimental duration.

### 2.4 Nutrient apparent digestibility

Following 28 d of the experimental phase, a digestibility test was conducted. This involved the collection of feed samples (Tifton hay, GAW, corn and soybean meal) as well as leftovers and fecal matter. Stool samples were directly collected from the rectal ampoule of the animals at specified intervals throughout the day (0, 2, 4, 6, 8 and 10 h post feeding), following the methodology outlined by Bispo et al. (2007). Subsequently, the samples were weighed, labeled and stored at - 15 °C . Upon completion of the collection period, the samples were homogenized to form composite samples and pre-dried in an oven with forced circulation at 65 °C for 72 h .

All feed samples, leftovers and fecal matter were finely ground using a Willey knife mill equipped with a 2.0 mm sieve to facilitate subsequent laboratory analyses. Fecal dry matter production (FDMP) was estimated by utilizing indigestible neutral detergent fiber (NDFi) as an internal marker. The determination of NDFi concentrations involved incubating samples comprising 1.0 g of concentrated feed and 0.5 g of hay, fecal matter and leftover diets within mini bags made of non-woven fabric (NWF) for 288 h in the rumen of a fistulated adult bovine male. Subsequently, the residual material from the incubation underwent digestion with neutral detergent, and the residue obtained was designated as NDFi following the INCT-CA method F/011/1, as outlined by Detmann et al. (2012).

FDMP was determined using the following formula: FDMP = indicator intake ( kg ) / indicator concentration in feces (%).

The digestibility coefficient (DC) of DM, organic matter (OM), crude protein (CP) and NDF was calculated using the following formula: DC = [( g of nutrient consumed - g of nutrient in feces) / ( g of nutrient consumed)] × 100. For feed conversion (FC), this was considered the total DMI divided by ADG.

### 2.5 Sample preparation and laboratory analysis

Samples of the provided feed were consistently collected prior to the formulation of experimental diets. The supplied feed, leftovers and fecal matter were collected over a period of 3 consecutive days to create composite samples. These composite samples were stored in a freezer at - 15 °C until further processing, including pre-drying, and subsequent analysis at a later time.

Pre-drying of the samples was conducted in an oven with forced air circulation at 65 °C for a duration of 72 h . Subsequently, the dried samples were pulverized in a mill fitted with a 1 mm mesh, followed by storage in appropriately labeled and hermetically sealed containers. These samples were then subjected to analysis for DM, OM, mineral material (MM), CP, ether extract (EE) and NDF. The analyses were performed according to the INCT-CA methods G-003/1, N-001/1, M-001/1, G-004/1, F-002/1 and F-004/1, respectively, as detailed in the methodologies outlined by Detmann et al. (2012).

To estimate the total carbohydrates (TC), the equation proposed by Sniffen et al. (1992) was employed, where TC = 100-(%CP+%EE+%Ashes) . For the estimation of non-fibrous carbohydrates (NFC), the equation recommended by Mertens (1997) was utilized, defined as NFC = 100-(%CP+%EE)+%MM+%NDF . Total digestible nutrients (TDN) were estimated following the equation described by Weiss (1999), expressed as TDN = DCP+DEE×2.25+DNFC+NDFcpD , where DCP represents digestible CP, DEE indicates digestible EE, DNFC signifies digestible NFC, and NDFcpD denotes digestible NDFcp. Here, DCP = (CP ingested - CP feces), DEE = (EE ingested - EE stool), DNFC = (NFC ingested - NFC stool), and NDFcpD = (NDFcp ingested - NDFcp stool).

Furthermore, to calculate the metabolizable energy (ME) content ( kcalMEperkgDM ), digestible energy (DE) was initially computed as the product of the TDN content and the 4.409/100 factor, considering the ME concentration to be 82 % of the DE (Silva and Leão, 1979).

The concentration of total tannins (TT) was determined by utilizing the butanol–HCl method as outlined by Terrill et al. (1992). Subsequently, the obtained result was converted to a percentage relative to the tannin content of black jurema. This conversion was achieved by employing a regression equation derived from the standard curve established using purified condensed tannin extracted from black jurema, in accordance with the methodology proposed by Beelen et al. (2006).

### 2.6 Economic analysis

Calculation of the effective operating cost (EOC) and total return (TR) across various treatments was performed. The EOC encompassed the procurement cost of lambs (USD 45.45 per head) as well as the cost of feed. The latter was determined by multiplying the unit value of each input by the quantity consumed in each diet. Average values per animal, reflecting 48 d of performance, were considered in this assessment.

As per Silva et al. (2009), the valuation of GAW involved considerations of transportation expenses, costs associated with the drying process of the raw residue, and its subsequent processing into mash form. The resulting valuation amounted to USD 0.33 per kilogram. Additionally, labor costs were factored in, assuming the requirement of an employee capable of dedicating 8 h to tasks such as animal feeding, facility maintenance and occasional medication administration. It was estimated that this individual could effectively handle the management of 300 animals per day, as outlined by Romão et al. (2017).

This expenditure was subsequently allocated on a per-animal basis. Labor remuneration was based on the prevailing minimum wage of USD 296.06 in 2017. Additionally, costs associated with anthelmintic application, vaccination and slaughtering were factored into the analysis. TR encompassed the values attributed to the entire carcass, excluding both carcass and skin components. Net profit (LL) was computed by determining the difference between TR and EOC.

### 2.7 Statistical analysis

The experimental design followed a completely randomized design comprising five treatments with eight replications each. Data underwent analysis of variance, and mean comparisons were conducted using the Tukey test at a significance level of 5 %. These analyses were performed utilizing the PROC general linear model procedure (GLM) procedure, in addition to regression analysis and orthogonal contrast implemented using the SAS^®^ program (SAS Institute Inc., 2003). Economic evaluations were carried out through spreadsheet calculations prepared in Microsoft Excel (version 2013) and were subjected to descriptive analysis for comparison.

## 3 Results

The DMI ( $g\;d^{-1}$ ) was not influenced ( p > 0.05) by the inclusion of GAW in the diet. However, the OMI was significantly higher with the inclusion of 6.93 % compared to the control treatment. The intake of EE showed an increasing linear effect ( p < 0.001) in which all the treatments were superior to the control diet. Thus, with 30 % GAW, the intake of EE presented an increase of 109.68 % in the control treatment.

**Table 2:** Intake of nutrients by Santa Inês lambs according to the inclusion levels of GAW in the diet.

Variable	Guava agroindustrial waste (%)					SEM	Linear	Quad
	0.0	7.5	15.0	22.5	30.0			
Dry matter intake								
$\mathrm{kg}\;d^{-1}$	1.23	1.36	1.27	1.40	1.34	0.14	0.121	0.422
Percent live weight	4.43	4.60	4.39	4.73	4.75	0.21	0.176	0.185
Per Kg W^0.75^(g)	101.6	107.2	101.8	110.2	109.4	5.80	0.111	0.543
Intake ( $g\;d^{-1}$ )								
Organic matter	1152^b^	1272	1198	1321	1272	0.13	0.069	0.447
Mineral	81.5	86.0	76.7	79.8	71.6	0.01	0.050	0.186
Crude protein	195.0	211.5	198.6	216.3	207.1	0.02	0.233	0.450
Ether extract	38.7^b^	50.0	57.5	73.7	81.2	0.01	< 0.0001^b^	0.894
Neutral detergent fiber	597.5	654.8	608.8	666.7	623.5	0.06	0.289	0.415
Total carbohydrates	918.7	1010.0	943.7	1033.7	986.2	0.10	0.205	0.413
Non-fibrous carbohydrates	322.5	352.5	336.2	366.2	351.2	0.03	0.111	0.405
Total digestible nutrients	1008.7	1092.5	1011.2	1092.5	1031.2	0.11	0.733	0.419

Means followed by different letters in the lines differ by Tukey's test at 5 % significance. SEM – standard error of the mean; Quad – quadratic. Intake of ether extract ( $g\;d^{-1}$ ). ^a^ y = 0.03850 + 0.00145x ( $R^{2}$ = 0.99); ^b^ orthogonal contrast: control vs. GAW inclusion levels.

**Table 3:** Digestibility of nutrients ( g per 100 g dry matter) according to the inclusion levels of GAW in the diet.

Variable	Guava agroindustrial waste (%)					SEM	Linear	Quad
	0.0	7.5	15.0	22.5	30.00			
Dry matter	60.7a^e^	61.0a	58.2ab	56.6b	55.5b	2.53	< 0.0001^a^	0.6132
Organic matter	61.8a^e^	61.7a	59.1ab	57.0b	55.8b	2.51	< 0.0001^b^	0.5736
Crude protein	69.8	71.3	71.0	70.6	73.3	2.90	0.0632	0.6043
Ether extract	75.9c^e^	77.8cb	77.0cb	80.4ab	83.4a	2.57	< 0.0001^c^	0.0700
Acid detergent fiber	52.8b^e^	56.7ab	58.3a	58.3a	61.4a	3.55	< 0.0001^d^	0.3404

Means followed by different letters in the lines differ by Tukey's test at 5 % significance. SEM – standard error of the mean; Quad – quadratic. ^a^ DM = 61.37250 - 0.19548x ( $R^{2}$ = 0.92); ^b^ OM = 62.41775 - 0.22013x ( $R^{2}$ = 0.95); ^c^ EE = 75.38500 + 0.23678x ( $R^{2}$ = 0.85); ^d^ NDF = 53.74850 + 0.26607x ( $R^{2}$ = 0.90); ^e^ orthogonal contrast: control vs. GAW inclusion levels.

The DM digestibility decreased linearly ( p < 0.001), while the NDF digestibility increased linearly ( p < 0.001) with the inclusion of GAW in the diet (Table 3), showing that the quality of GAW fiber was 16.47 % more digestible at the level of 30 % inclusion when compared to the treatment that only obtained Tifton hay.

**Table 4:** Growth performance and feed conversion of Santa Inês lambs according to the inclusion levels of GAW in the diet.

Variable	Guava agroindustrial waste (%)					SEM	Linear	Quad
	0.0	7.5	15.0	22.5	30.0			
Initial weight ( kg )	21.3	21.7	21.2	21.5	20.7	2.74	0.672	0.646
Final weight ( kg )	34.2^c^	37.3	36.8	37.7	35.9	3.28	0.331	0.059
Total weight gain ( kg )	12.9^c^	15.5	15.6	16.1	15.1	2.61	0.103	0.055
Average daily gain ( g )	269.6^c^	329.8	327.2	338.0	317.1	0.05	0.099	0.037^a^
FC ( kgDMperkgADG )	4.7^c^	4.2	3.9	4.1	4.3	0.65	0.224	0.033^b^

Means followed by different letters in the lines differ by Tukey's test at 5 % significance. FC – feed conversion; SEM – standard error of the mean; Quad – quadratic. ^a^ ADG, y = 274.50 + 7.05 x - $0.19x^{2}$ ( $R^{2}$ = 0.90). ^b^ FC, y = 4.73 - 0.08 x + $0.00238x^{2}$ ( $R^{2}$ = 0.92). ^c^ Orthogonal contrast: control vs. GAW inclusion levels.

FLW, TWG, ADG and FC showed orthogonal contrast ( p < 0.05), in which the animals that ingested the control diet had lower performances (Table 4). The FC also had a quadratic effect ( p < 0.05), decreasing when GAW was included in the diet. The animals with GAW added to the diet obtained greater weight gains, which is reflected in a better feed conversion and shows that GAW had a positive influence on the growth parameters, regardless of the level of inclusion in the diet.

**Table 5:** Cost–benefit ratio of feeding Santa Inês lambs with guava agroindustrial waste.

	Guava agroindustrial waste (%)				
	0.0	7.5	15.0	22.5	30.0
Diet cost ( kgperdiet , USD)	0.33	0.31	0.28	0.26	0.24
Whole housing					
Quantity ( kg )	112.82	130.32	126.68	125.13	129.87
Price (USD)	4.55	4.55	4.55	4.55	4.55
Non-carcass constituents					
Quantity ( kg )	54.42	58.25	55.94	58.73	56.22
Price (USD)	2.27	2.27	2.27	2.27	2.27
Skin					
Quantity ( kg )	8	8	8	8	8
Price (USD)	1.82	1.82	1.82	1.82	1.82
Effective operating cost (USD)	547.39	551.52	530.82	532.33	515.04
Gross income (USD)	651.05	739.28	717.51	716.81	732.65
Gross margin (USD)	103.66	187.76	186.68	184.48	217.61
Return rate (USD)	0.06	0.10	0.11	0.11	0.13
Safety margin (%)	4.82	7.70	7.88	7.80	9.00
Cost benefit (USD per day)	0.36	0.41	0.41	0.41	0.43

Regarding the parameters of the economic analysis (Table 5), there was a positive gross margin for all the diets, showing the highest margin for the diet that contained 30 % GAW, USD 217.61, due to the lower EOC in this treatment. The rate of return showed linear growth when GAW was included in the diet; i.e., for each dollar (USD 1.00) applied, USD 0.06 (0 % GAW) was achieved at a USD 0.13 (30 % GAW) return.

## 4 Discussion

The improved productive performance values observed in sheep fed with GAW can be attributed to the superior fiber quality of this by-product. As illustrated in Table 1, increasing the inclusion of GAW in the lambs' diet led to a reduction in the proportion of Tifton hay, resulting in higher fiber digestibility. Additionally, there was a significant increase in the digestibility of crude protein, ether extract and acid detergent fiber, which may have enhanced feed conversion and, consequently, productive performance.

On the other hand, a reduction in the digestibility of dry matter and organic matter was observed, likely due to the higher lignin content associated with the inclusion of GAW. However, these changes were not significant enough to impact nutrient intake (Table 2).

The average DMI observed in this experiment aligns with the recommendations outlined in the NRC (2007) guidelines for animals weighing approximately 40 kg , where intake typically ranges around 3.31 % of body weight, resulting in daily gains of 250 g . Notably, the average DMI of 4.58 % of LW observed with the inclusion of GAW suggests that the heightened levels of fiber and phenolic compounds, including lignin and tannins, associated with GAW incorporation are akin to findings reported by Lousada Júnior et al. (2005). In their study, DMI values of 4.4 % LW and 106.8 $g\;\mathrm{DM}\;{\mathrm{kg}}^{-1}\;{\mathrm{LW}}^{0.75}$ were documented. Moreover, the intake of MM, CP, NDF, TC, NFC and TDN exhibited no significant differences across the treatments. This similarity in intake patterns was attributed to the comparable nutrient composition observed between the evaluated ingredients, i.e., GAW and Tifton hay.

The enhanced digestibility of NDF attributed to the inclusion of GAW is primarily ascribed to its low ADF content of approximately 0.69 % and the nitrogen content within NDF of approximately 0.45 %. These indicators suggest heightened availability of nitrogen for rumen microorganisms when GAW is introduced into the diet (Braga et al., 2016). However, as GAW inclusion levels reach 20 %, a decline in DM digestibility is noted. This phenomenon is attributed to elevated levels of lignin and ADF in the diet, impeding microbial degradation and subsequently diminishing diet degradability and digestibility coefficients (Braga et al., 2016). Notably, Hassan et al. (2016) reported a DM digestibility of 60.67 % upon incorporating up to 20 % GAW in sheep diets.

Given that the diets were formulated to maintain isoprotein levels (Table 1), with soybean meal representing the primary protein source at 53 %, it appears that tannin levels within the diets were insufficient to induce significant protein complexation, thereby resulting in reduced digestibility. Furthermore, the presence of vitamin C may have contributed to hindering oxidative protein denaturation (Seven, 2008). Bikrisima et al. (2014) noted that optimal performance correlates with vitamin C and antioxidant content, suggesting potential beneficial effects in this regard. Furthermore, GAW has a favorable amino acid profile that is notably rich in arginine, glutamic acid, aspartic acid, glycine and leucine (Habib, 1986). This profile may account for the increased protein digestibility in diets containing GAW, even without changes in total protein intake. Moreover, GAW contains 12 types of grains, which significantly enhances its nutritional value for feeding purposes (Aly, 1981).

The ADG observed in treatments incorporating GAW exhibited a notable increase (31.2 %). However, this disparity can be elucidated by the higher DM recorded in the present experiment compared to the estimations provided by the NRC (2007). Additionally, the elevated energy content, particularly EE, associated with GAW could have contributed to this phenomenon, considering that the feed conversion ratio across the treatments stood at 4.15 kg of dry matter per kilogram of ADG.

The outcomes of this study are in line with those reported by Hassan et al. (2016), who concluded that GAW can be integrated into lamb diets at the 20 % level without any detrimental effects on the final live weight, weight gain and feed conversion ratio. Conversely, Silva et al. (2014) observed a higher feed conversion ratio of 7.91 with the inclusion of 24.6 % GAW in the diet, albeit without processing. Furthermore, Costa et al. (2020) noted an inverse relationship between the final lamb weight and GAW levels, suggesting that increasing the percentage of GAW in the diet, in lieu of corn, may have an adverse impact on the final lamb weight.

The costs associated with each diet experienced a decline following the substitution of Tifton hay with GAW, owing to the relatively lower cost of the residue compared to hay. The initial price of the control diet stood at USD 0.33 per kilogram, with each successive level of GAW incorporation resulting in a reduction of USD 0.02 per kilogram in the diet's price. This reduction persisted until it reached the 30 % GAW inclusion level, where the price of the diet exhibited a difference of USD 0.09 per kilogram compared to the control treatment. This represents a 27 % variance from the control treatment to the level with the highest GAW content in the diet.

The treatment featuring 7.5 % GAW exhibited the highest feed cost, consequently leading to a greater EOC of USD 551.52. Nonetheless, as the proportion of GAW in the diet increased, the EOC decreased. This trend mirrors the findings reported by Hassan et al. (2016), who observed a comparable pattern when investigating GAW inclusion in sheep diets, concluding that the performance was optimal up to the 20 % inclusion level.

Based on the revenue derived from the sale of the entire carcass, excluding its constituents, it was observed that sheep consuming diets containing GAW exhibited an increase in carcass yield. Specifically, diets containing 7.5 % and 30 % GAW inclusion demonstrated the highest carcass weights, totaling 130.32 and 129.87 kg , respectively. These figures represent a 15 % increase compared to the control treatment. Additionally, edible components beyond the carcass, such as the heart, liver, lung, spleen, stomach, intestines, kidneys, brain and blood, were found in greater quantities with the incorporation of GAW into the diet. This trend is likely due to the high EE content of the residue, which facilitates heightened metabolic activity and consequently leads to increased organ size.

The economic analysis parameters revealed a positive gross margin across all the diets, with the highest margin observed in the diet containing 30 % GAW, amounting to USD 217.61. This significant margin increase can be attributed to the lower EOC associated with this treatment. Specifically, the market price per kilogram of meat could potentially decrease by up to 29.70 % in the diet containing 30 % GAW. Furthermore, the cost–benefit ratio was favorable (positive), indicating that, for every dollar (USD 1.00) invested, the return amounted to USD 0.43. In light of these findings, the incorporation of up to 30 % GAW in the diet has emerged as the optimal economic choice. This was largely due to the reduced expenses associated with Tifton hay and the observed increases in carcass weights, ultimately contributing to enhanced profit margins.

## 5 Conclusions

Guava agroindustrial waste (GAW) demonstrates effective utilization as a feed ingredient for sheep, with optimal inclusion levels observed for up to 30 % of the diet.

## Data Availability

No data sets were used in this article.
